# Efficacy and safety of therapy of chronic hepatitis B with telbivudine (LdT) in patients with HIV-infection without HAART

**DOI:** 10.1186/1758-2652-13-S4-P209

**Published:** 2010-11-08

**Authors:** SP Tsarenko, AV Kravchenko, VG Kanestri, SL Maximov

**Affiliations:** 1 Moscow State University of Medicine & Dentistry, 4-15, 8-ya str/ Sokolinoy Gory, Moscow, Russian Federation; 2 Russian Federal AIDS Center, Moscow, Russian Federation

## Introduction

According to the European recommendations (version 5.2., November 2009) at patients with the HIV-infection and chronic hepatitis B (CHB), not receiving HAART, adefovir and LdT may be used as an alternative regime. In the Russian Federation adefovir is not registered and tenofovir was registered only in March 2010.

## Objective

To study efficacy and safety Ltd at patients with HIV-infection and CHB (HIV/CHB).

## Methods

12 patients with HIV/CHB (men 10), middle age of 33,5 years, without HAART (CD4 + - 450-650 cells/mm3), since 2009 received LdT (600 mg QD). All patients had HBsAg, HBV DNA. 9 from 12 patients had HBeAg. Duration of treatment has made 3-12 months.

## Summary of results

At all patients had the positive result of therapy with LdT: 7 patients had disappearance of HBV DNA in 1-6 months; at 1 patient after 3 months of therapy observed disappearance of HBsAg and appearance anti-HBs and Ltd has been cancelled. At 5 patients through 6-9 months of treatment is marked a decreasing of level of HBV DNA on 2-3 log IU/ml. At 4 from 9 patients with HBeAg observed seroconversion to anti-HBe. In absence HAART registered fluctuations of level of HIV RNA was not statistically significant. The adverse events of therapy with Ltd have not been registered.

## Conclusions

Therapy of CHB with Ltd at patients with HIV-infection was effective: at 58,3 % of patients had disappearance of HBV DNA, and at 4 from 9 patients with HBeAg observed seroconversion. Influence of therapy with Ltd on dynamics of HIV RNA it wasn’t revealed.

